# Transcriptomic Insights into Sre1-Related Regulatory Responses to Hypoxia, Cobalt Chloride, and Clotrimazole in *Phaffia rhodozyma*

**DOI:** 10.3390/jof12030200

**Published:** 2026-03-10

**Authors:** Marcelo Baeza, María Soledad Gutiérrez, Melissa Gómez, Jennifer Alcaíno

**Affiliations:** Departamento de Ciencias Ecológicas, Facultad de Ciencias, Universidad de Chile, Santiago 7800003, Chile; mbaeza@uchile.cl (M.B.); soleguti@uchile.cl (M.S.G.); melissa.gomez@ug.uchile.cl (M.G.)

**Keywords:** SREBP, Sre1, hypoxia, cobalt chloride, clotrimazole, RNA-seq, *Phaffia rhodozyma*

## Abstract

Sterol regulatory element-binding proteins (SREBPs) are transcription factors that regulate lipid homeostasis and have been associated with hypoxia adaptation in fungi. In the yeast *Phaffia rhodozyma*, the SREBP ortholog named Sre1 regulates sterol biosynthesis, but its contribution to stress-responsive transcriptional programs remains poorly understood. We performed RNA-seq analyses to evaluate the transcriptional responses of wild-type (WT) and ∆*sre1* mutant strains exposed to hypoxia, cobalt chloride (CoCl_2_), and clotrimazole treatments. Differentially expressed genes (DEGs) were analyzed using KEGG mapping to assess the treatment-induced transcriptional changes in both strains and to evaluate the potential contribution of Sre1 to these responses. In the WT strain, hypoxia induced the most extensive transcriptional changes, while CoCl_2_ elicited a moderate response partially overlapping with hypoxia. Downregulated DEGs predominated in both conditions, and all CoCl_2_-associated KEGG pathways were also identified under hypoxia. In contrast, the Δ*sre1* mutant showed an increased number of DEGs in response to clotrimazole and CoCl_2_, with most clotrimazole-responsive genes being mutant-specific, indicating distinct Sre1-associated transcriptional responses under these conditions. Shared downregulated DEGs under CoCl_2_ and hypoxia suggest that basal Sre1 activity may contribute to modulation of gene expression programs related to core cellular processes. However, Sre1-dependent regulation alone did not account for the extensive transcriptional reprogramming observed under the applied hypoxic treatment.

## 1. Introduction

*Phaffia rhodozyma* (formerly *Xanthophyllomyces dendrorhous*) is a basidiomycete yeast best known for its ability to synthesize astaxanthin, a carotenoid of high industrial relevance due to its antioxidant properties and applications in aquaculture, cosmetics, and biotechnology [1,2,3]. As one of the few organisms that produces astaxanthin de novo, *P. rhodozyma* has become an important model for studying carotenoid biosynthesis and its regulation [4]. In nature, this yeast is typically found in cold habitats and oxidative or UV-exposed environments [5,6], and astaxanthin accumulation has been proposed to contribute to its tolerance under such conditions [7,8]. However, the molecular mechanisms that connect environmental stimuli to transcriptional regulation and stress-responsive pathways in *P. rhodozyma* remain poorly understood.

Environmental and chemical perturbations provide valuable tools for dissecting stress-responsive pathways in fungi. Among these, oxygen availability is a major factor influencing cellular physiology. Aerobic organisms rely on mitochondrial respiration for efficient energy production. Because oxygen levels fluctuate across natural habitats and lifestyles, fungi have evolved mechanisms to detect and respond to oxygen limitation [9,10]. These mechanisms are crucial for adapting to hypoxic conditions that may arise during growth on diverse substrates or within host environments [11]. Research in this field has largely focused on pathogenic fungi, where hypoxia responses contribute to virulence [12,13]. Oxygen levels can be sensed directly through oxygen-sensing proteins or ligands, or indirectly through changes in cellular homeostasis, such as alterations in redox balance [13]. Sterol metabolism also functions as an oxygen-sensing system, as several steps of lipid biosynthesis require molecular oxygen. For example, in *Candida albicans*, oxygen limitation induces the transcription of genes involved in ergosterol biosynthesis and fatty acid metabolism, highlighting the central role of oxygen in lipid pathway regulation [14,15,16]. Cobalt chloride (CoCl_2_) is widely used as a chemical hypoxia mimetic [17]. In *Saccharomyces cerevisiae*, cobalt interferes with oxygen-dependent Fe-containing enzymes, including diiron-oxo enzymatic steps involved in sterol synthesis and fatty acid desaturation, as well as 4Fe-4S dehydratase enzymes of the mitochondria [18]. In *Cryptococcus neoformans*, CoCl_2_ disrupts several steps of ergosterol biosynthesis and induces a transcriptional response that only partially overlaps with that triggered by low oxygen, indicating that although CoCl_2_ and hypoxia share some consequences, they also affect cellular physiology in distinct ways [17]. Clotrimazole likewise perturbs sterol metabolism, but through a different mechanism. As an azole antifungal, it inhibits the cytochrome P450 lanosterol 14-alpha demethylase (Erg11), an essential oxygen-dependent enzyme of the ergosterol pathway [19]. Inhibition of Erg11 reduces ergosterol levels, leading to the accumulation of toxic sterol intermediates that compromise membrane integrity [20]. This triggers compensatory transcriptional and metabolic responses to restore sterol homeostasis [21]. Together, hypoxia, CoCl_2_, and clotrimazole perturb overlapping but mechanistically distinct aspects of fungal metabolism, making them suitable for comparative analyses of transcriptional regulation linked to sterol homeostasis and cellular stress.

A central regulator expected to respond to these perturbations is the sterol-regulatory element–binding protein (SREBP) family. SREBPs are ER-membrane-bound transcription factors that regulate lipid homeostasis by regulating genes of enzymes involved in sterol, fatty acid (FA), triacylglycerol, and phospholipid biosynthesis [22]. In fungi, SREBPs coordinate sterol metabolism and contribute to adaptation under restrictive environmental conditions. The best-characterized fungal SREBP pathway is that of the fission yeast *Schizosaccharomyces pombe*, where the SREBP ortholog named Sre1 is activated under hypoxia and sterol depletion and is essential for the transcriptional induction of genes required for adaptation to low-oxygen conditions [23,24]. In *S. pombe*, CoCl_2_ and ergosterol biosynthesis inhibitors, such as clotrimazole, induced an Sre1-dependent reporter, whereas the Δ*sre1* mutant failed to induce these responses [25], reflecting the activation of Sre1 by the treatments. In the basidiomycete pathogen *C. neoformans*, Sre1 is similarly required for adaptation to hypoxia and for survival during azole treatment [26]. In *Aspergillus fumigatus*, hypoxia induces proteolytic activation of the SREBP-like transcription factor SrbA, which is required for ergosterol biosynthesis, azole drug resistance, and virulence [27,28,29]. Loss of SrbA results in defective growth under hypoxia and causes extreme susceptibility to triazoles [30]. SrbA also contributes to the transcriptional remodeling that occurs during hypoxia [31], underscoring the conserved role of fungal SREBPs in coordinating lipid metabolism and responding to environmental stress. In plant pathogenic fungi, SREBP homologs have also been implicated in adaptation to host-associated environments. In the rice blast fungus *Magnaporthe oryzae*, the SREBP homolog *MoSre1* is induced under hypoxic conditions and contributes to sterol biosynthesis and invasive growth during infection, although it was not shown to be strictly required for disease severity [32]. More recently, functional analysis of Sre1 in the necrotrophic pathogen *Botrytis cinerea* demonstrated that Sre1 is required for proper ergosterol biosynthesis, hypoxia adaptation, and virulence, highlighting a conserved role for fungal SREBPs in coordinating lipid homeostasis, development, and pathogenicity in plant–fungus interactions [33]. In *P. rhodozyma*, sterol homeostasis is also regulated by the SREBP homolog Sre1 [34], which additionally influences carotenoid biosynthesis by regulating genes of the mevalonate pathway and of key carotenogenic enzymes [35,36]. Heterologous expression of the N-terminal domain of Sre1 (Sre1N) from *P. rhodozyma* partially rescued the hypoxic growth defect of a *S. pombe* Δ*sre1* mutant, and Δ*sre1* mutants of *P. rhodozyma* failed to grow in the presence of CoCl_2_ or clotrimazole [34], suggesting a conserved role of Sre1 from *P. rhodozyma* that contributes to oxygen-responsive signaling and sterol homeostasis.

Studies across fungi show that SREBPs act as conserved regulators that integrate signals derived from low-oxygen conditions, CoCl_2_ exposure, and azole inhibition. These signals converge on sterol homeostasis and stress adaptation, positioning SREBPs as key regulators of transcriptional responses induced by these perturbations. While SREBP function and lipid-based oxygen sensing have been characterized in pathogenic fungi, this has not been explored in *P. rhodozyma*. Accordingly, this study was designed as a comparative and exploratory transcriptomic analysis aimed at capturing early transcriptional responses to these perturbations and to define the contribution of Sre1 at a global level. Consistent with this approach, we analyzed the transcriptomic responses of *P. rhodozyma* exposed to short-term hypoxia, CoCl_2_, and clotrimazole treatments, including comparisons with a Δ*sre1* mutant. 

## 2. Materials and Methods

### 2.1. Strains and Culture Conditions

The *P. rhodozyma* strains used in this work were the wild-type (WT) CBS 6938 (ATCC 96594) and the mutant CBS.*sre1^−^* (hereafter referred to as Δ*sre1* mutant), in which approximately 90% of the coding region of the *SRE1* gene was replaced with a zeocin resistance cassette [34]. Strains were grown at 22 °C with constant agitation in YM medium (0.3% yeast extract, 0.3% malt extract, and 0.5% peptone) supplemented with 1% glucose. For all RNA-seq analyses, three biological replicates were included for each strain and condition. Cultures were grown for 36 h (exponential growth phase) under normoxic conditions (21% O_2_) to ensure metabolically active cells, and were subsequently exposed for 4 h to the following treatments: (i) hypoxia (2–3% O_2_, in an Anaerobic Chamber, Coy Lab Products, Grass Lake, MI, USA), (ii) supplementation with CoCl_2_ (400 μM), (iii) supplementation with clotrimazole (0.1 μg/mL), or (iv) a control condition maintained under normoxia without chemical supplementation. This experimental design was chosen to evaluate early transcriptional responses (i.e., within a single *P. rhodozyma* generation time) under metabolically active conditions, as the Δ*sre1* mutant cannot grow in the presence of CoCl_2_ or clotrimazole [34]. Identical conditions were applied to both strains (WT and Δ*sre1* mutant) to allow direct comparisons.

### 2.2. RNA Purification and RNAseq Analysis

RNA extraction was carried out according to Chomczynski and Sacchi [37]. Briefly, 5 mL of yeast culture was centrifuged at 4000× *g* for 5 min, the cellular pellet was resuspended in 200 μL of lysis buffer (0.02 M sodium acetate, pH 5.5; 0.5% SDS; 1 mM EDTA), and 100 μL of 0.5 mm glass beads (BioSpec Products Inc., Bartlesville, OK, USA) were added. The suspension was homogenized for 1 min using a Mini-Beadbeater 16 (BioSpec Products Inc., Bartlesville, OK, USA). Then, 800 μL of TRI Reagent (Ambion^TM^, Thermo Fisher Scientific Inc., Waltham, MA, USA) was added, the mixture was homogenized again for 1 min and then incubated on ice for 10 min. Next, 200 μL of chloroform was added, the mixture was manually shaken for 15 s, incubated at room temperature for 6 min, and centrifuged at 18,000× *g* for 10 min at room temperature. The aqueous phase was recovered, and one volume of cold isopropanol and half a volume of precipitation solution (1.2 M NaCl, 0.8 M sodium citrate) were added. The mixture was incubated for 1 h at −20 °C and then centrifuged at 18,000× *g* for 10 min. The RNA pellet was washed with 1 mL of 70% ethanol and centrifuged at 18,000× *g* for 5 min. The supernatant was carefully removed, the pellet was dried at 37 °C, and the RNA pellet was resuspended in 30–60 μL of nuclease-free Milli-Q water and stored at −80 °C until analysis. RNA concentration was measured spectrophotometrically using the Epoch 2 system with a Take3 microplate and Gene5 software (version 2.09) (BioTek Instruments, Winooski, VT, USA). RNA purity was evaluated by the A_260_/A_280_ absorbance ratio, and RNA integrity was assessed using the BioAnalyzer 2100 system (Agilent, Santa Clara, CA, USA).

Library construction and high-throughput sequencing were performed by the National Center for Genomic Analysis (Barcelona, Spain). Briefly, libraries were prepared using the TruSeq Stranded mRNA kit (Illumina Inc., San Diego, CA, USA) with a target fragment size of 75 bp, and sequencing was conducted as paired-end reads on the HiSeq 2000 system (Illumina Inc., San Diego, CA, USA).

Raw paired-end RNA-Seq reads were quality-filtered and adapter-trimmed using fastp v0.23.2 [38]. For each library, the average read length was estimated, and the minimum read length threshold was set adaptively according to sequencing depth (50 bp for 150 bp reads, 40 bp for 100 bp reads, and 30 bp for 75 bp reads). Low-quality bases with a Phred score < 20 were trimmed, and reads failing to meet this threshold were discarded. Poly-G and poly-X tails were removed to eliminate sequencing artifacts. Paired reads were synchronized to maintain mate consistency, and adapter sequences were removed. Quality control metrics are summarized in Supplementary Table S1.

RNA-Seq reads were aligned to the *P. rhodozyma* CBS 6938 reference genome (GenBank accession: GCA_014706385.1) using previously annotated gene information [35,39], with Rsubread v2.18.3 [40]. The resulting BAM files were then sorted, indexed, and filtered to retain high-quality, properly paired reads using functions from the Rsamtools package [41,42]. Gene-level counts were generated using featureCounts with GFF annotation, and expression values were normalized to RPKM and TPM for each gene in each sample, resulting in matrices of raw counts and normalized expression for downstream analyses. Reproducibility across biological replicates was assessed using Pearson and Spearman correlations on log_2_(TPM + 1)-transformed expression values. All biological replicates showed high reproducibility with Pearson and Spearman correlation coefficients ranging from 0.93 to 1.00 and from 0.92 to 1.00, respectively. Differential gene expression analysis was performed using DESeq2 v1.40.1 [43] in R. Raw gene-level counts were imported from the count matrix generated by featureCounts. Sample conditions were assigned based on the first character of each sample name, and all pairwise comparisons between conditions were conducted. For each comparison, a DESeqDataSet was constructed, with the design formula ~ Condition, and the reference condition was specified to ensure consistent contrast direction. The DESeq2 workflow was executed to estimate size factors, dispersion, and negative binomial model parameters, followed by Wald tests to identify differentially expressed genes. Results for each condition pair are shown in Supplementary Table S2.

KEGG’s pathway analysis was performed using the KEGG Automatic Annotation Server (KAAS) [44], applying default GHOSTX parameters [45] and eukaryotic gene datasets. Plots were generated in Python 3 using pandas (version 2.2.3) [46], Matplotlib (version 3.9.2) [47], seaborn (version 0.13.2) [48], and NumPy (version 2.1.3) [49] libraries.

## 3. Results and Discussion

### 3.1. Transcriptomic Profiling of P. rhodozyma Under Hypoxia, CoCl_2_, and Clotrimazole Treatments

**Figure 1:**
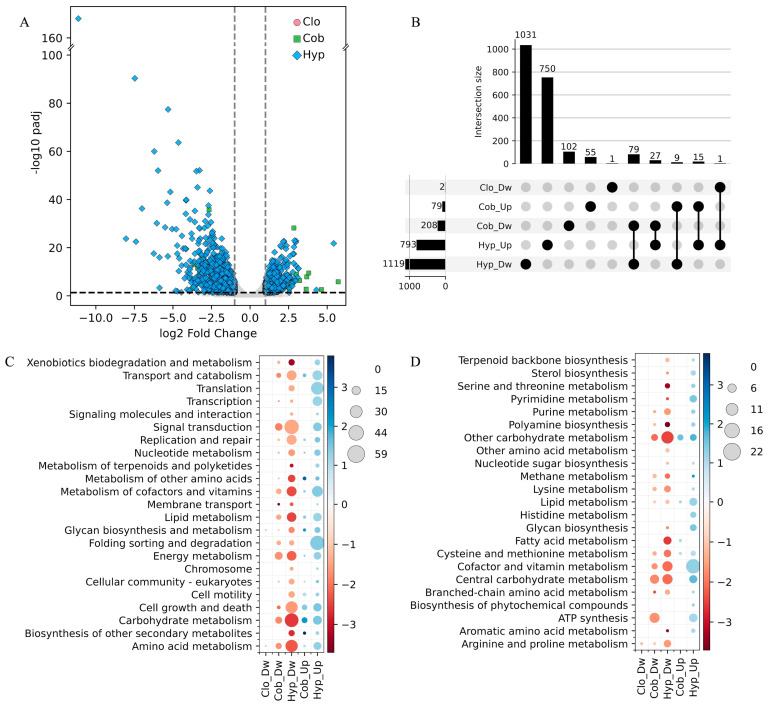
Comparative analysis of transcriptional response of *P. rhodozyma* to different treatments. The transcriptomes of the wild-type strain under hypoxic (Hyp), CoCl_2_ (Cob), and clotrimazole (Clo) treatments were compared to those of the untreated control. (**A**) Volcano plot showing differentially expressed genes (DEGs). Significance thresholds are indicated by dashed lines (|log_2_FC| ≥ 1.0, *p*adj ≤ 0.05). (**B**) Upset plot displaying exclusive and overlapping down-regulated (Dw) and up-regulated (Up) DEGs among treatments. Classification of DEGs with KEGG pathways (**C**) and functional modules (**D**). Circle size represents the number of DEGs, and circle color indicates the average log_2_FC.

Differentially expressed genes (DEGs) in the wild-type (WT) strain of *P. rhodozyma* exposed to hypoxia, CoCl_2_, and clotrimazole were identified by comparing transcriptomes obtained from treated cultures with the untreated control. The strongest response was observed under hypoxia, where the number of DEGs was 6.7-fold higher than in the CoCl_2_ treatment (Figure 1A,B). In both conditions, the number of downregulated DEGs (DwDEGs) exceeded that of upregulated ones (UpDEGs), by 1.4-fold under hypoxia and 2.6-fold under CoCl_2_. Only two DEGs, both downregulated, were detected in response to clotrimazole (Figure 1A,B). Comparison of the hypoxia and CoCl_2_ treatment responses revealed substantial divergence. Overall, 92% of the DwDEGs and 95% of the UpDEGs identified under the hypoxic treatment did not match those found under the CoCl_2_ treatment, whereas 49% of the DwDEGs and 70% of the UpDEGs in CoCl_2_ were exclusive to this treatment. Among the DEGs common to both treatments, 79 were DwDEGs, 15 were UpDEGs, and 36 DEGs showed opposite regulation. These results indicate substantial differences in the transcriptional responses of *P. rhodozyma* to these treatments, with hypoxia triggering the strongest response and CoCl_2_ producing a more moderate response that partially overlaps with that of hypoxia.

To further characterize the transcriptomic changes in response to the treatments, the DEGs were classified into KEGG metabolic pathways and functional modules (Figure 1C,D). Considerable differences were observed among treatments in both the number of identified KEGG pathways and modules, and the number of DEGs in each category. The two DwDEGs detected under clotrimazole treatment were assigned to the Amino acid metabolism and Glycan biosynthesis and metabolism pathways, and the Arginine and proline metabolism module. Six pathways and nine modules among DwDEGs were found exclusively under hypoxia, among which the Cell motility, Translation, and Biosynthesis of other secondary metabolites pathways, as well as modules related to Fatty acid metabolism and Serine and threonine metabolism, contained the highest numbers of DwDEGs. Similarly, nine pathways and 17 modules among UpDEGs were exclusive to hypoxia treatment, where the Folding sorting and degradation, Translation, and Transcription pathways and the modules Arginine and proline metabolism, Aromatic amino acid metabolism, and ATP synthesis contained the highest numbers of UpDEGs within the hypoxia-exclusive set. All pathways and modules detected under CoCl_2_ exposure were also detected in hypoxia. In general, the number of DEGs associated with each category was higher under the hypoxic treatment. The largest differences regarding the DwDEGs were observed in the Signal transduction, Carbohydrate metabolism, and Cell growth and death pathways, as well as in the ATP synthesis, Other carbohydrate metabolism, Cofactor and vitamin metabolism, and Fatty acid metabolism modules. The most pronounced differences among the UpDEGs were observed in the Metabolism of cofactors and vitamins, Transport and catabolism, and Lipid metabolism pathways, and the Lipid metabolism module. In general, our results show down- and upregulation of cellular processes under hypoxic and CoCl_2_ treatments, consistent with observations in other yeasts and fungi. Previous studies have reported upregulation of lipid and fatty acid metabolism under hypoxia in *C. albicans* [50] and of genes involved in sterol biosynthesis in *C. albicans* and *A. fumigatus* [51]. Downregulation of genes associated with fatty acid degradation was reported in *C. neoformans* [52], and upregulation under CoCl_2_ treatment of genes related to DNA synthesis and repair in *Debaryomyces hansenii* [53].

Overall, the observed transcriptomic changes in *P. rhodozyma* indicate distinct magnitudes of transcriptional responses under clotrimazole, CoCl_2_, and hypoxia treatments under the tested conditions, with clotrimazole inducing minimal changes, CoCl_2_ eliciting a moderate response, and hypoxia triggering the most extensive transcriptional remodeling. Interestingly, the CoCl_2_ transcriptional response partially overlaps with that induced by hypoxia.

### 3.2. Comparison of Transcriptomic Responses Between Hypoxia, CoCl_2_, and Clotrimazole Treatments

**Figure 2:**
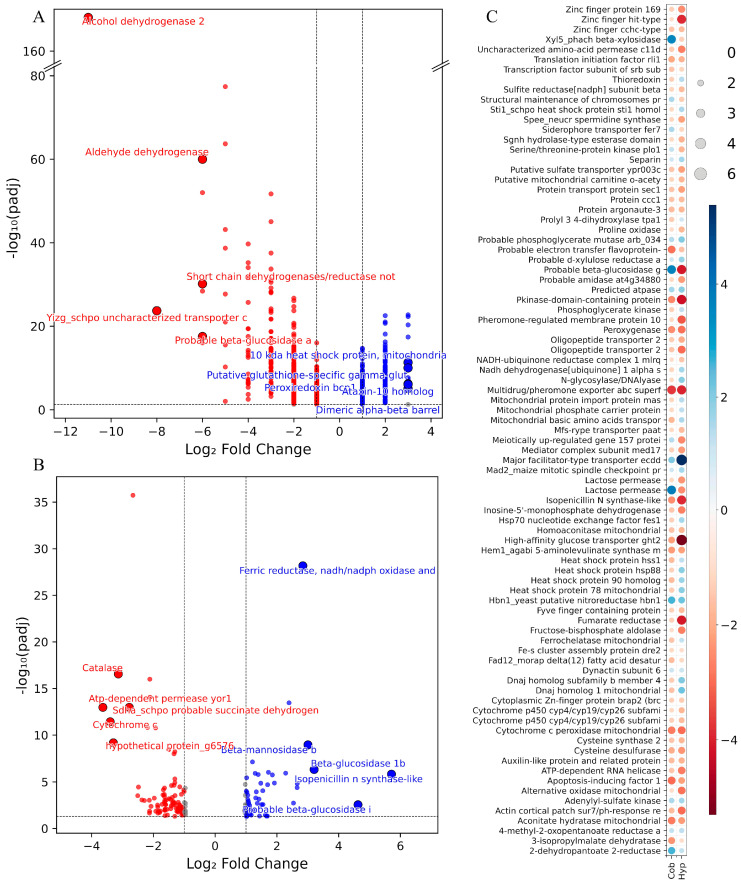
Highly differentially expressed genes in *P. rhodozyma* under hypoxic and CoCl_2_ treatments. Volcano plots show genes exclusively regulated under (**A**) hypoxia (Hyp) and (**B**) CoCl_2_ (Cob) treatments, with the top five up- and downregulated genes labeled. Significance thresholds are indicated by dashed lines (|log_2_FC| ≥ 1.0, *p*adj ≤ 0.05). (**C**) Heatmap of genes commonly regulated under both treatments: log_2_FC value (circle color) and |log_2_FC| value (circle size).

The DEGs identified by BLASTp (version 2.17.0) searches against the NCBI databases were classified according to whether they were observed exclusively under hypoxia, under CoCl_2_ treatment, or under both treatments (Figure 2). Among the 655 downregulated genes exclusively observed under hypoxia, the five with the strongest downregulation were Alcohol dehydrogenase 2, Yizg_schpo uncharacterized transporter c1002.16c, Probable beta-glucosidase a, Aldehyde dehydrogenase, and Short chain dehydrogenases/reductase notp’, a pattern that contrasts with previous reports. For example, aldehyde dehydrogenase, which, together with long-chain acyl-CoA dehydrogenase, participates in the detoxification of aldehydes formed during anaerobic metabolism or oxidative stress, is induced in *Paracoccidioides* under short-term hypoxia [54]. Similarly, the induction of three intracellular β-glucosidase genes in response to oxygen depletion was described in *Phlebia radiata* [55]. Besides the carbon source, fungal alcohol dehydrogenase (*ADH*) genes are also regulated by oxygen level. For example, in *Scheffersomyces stipitis* (formerly *Pichia stipitis*) cultured on xylose, *ADH1* expression increased when cells were shifted to oxygen-limited conditions [56,57]. Moreover, the *ADH2* promoter from this yeast has been utilized to drive protein expression in *Pichia pastoris* under microaerobic conditions [58] and has also been engineered for enhanced performance under these conditions [59]. However, in *S. cerevisiae*, both the RNA and protein levels of *ADH2* decreased as oxygen availability decreased [60], which is consistent with our observations. Overall, our results suggest that the products of the top five hypoxia-responsive DwDEGs are not essential for the initial response to hypoxia in *P. rhodozyma*. However, it is probable that their expression may be induced following prolonged hypoxic exposure as part of a secondary or delayed hypoxia-regulated response.

Of the 539 exclusively upregulated genes under the hypoxic treatment, the top five corresponded to 10 kDa heat shock protein mitochondrial, Heat shock protein 60 mitochondrial, D-arabinitol dehydrogenase 1, Hsp90 co-chaperone Aha1, and Extracellular metalloprotease mgg_08041. The upregulation of these genes likely reflects an early broad transcriptional response associated with metabolic adjustment and stress-related pathways under hypoxic conditions. Mitochondrial chaperones are involved in proper protein folding within mitochondria, which is crucial for energy production under oxygen-limited conditions [61]. The Hsp90 co-chaperone Aha1 enhances the activity of Hsp90, a molecular chaperone required for folding and stabilization of a wide range of substrate proteins [62]. Among metabolic enzymes, D-arabinitol dehydrogenase 1 primarily produces D-arabinitol, a compound that quenches reactive oxygen species in *Uromyces faba* [63], while extracellular metalloproteases contribute to nutrient acquisition from the environment, and have been implicated in stress-associated processes [64].

A total of 62 genes were downregulated exclusively under CoCl_2_ treatment, with the five most strongly repressed corresponding to ATP-dependent permease yor1, Cytochrome c, Catalase, Sdha_schpo probable succinate dehydrogenase[ubiquinone] flavoprotein subunit mitochondrial, and Efflux pump hime. On the other hand, 50 genes were exclusively upregulated under CoCl_2_, with the top five including Isopenicillin N synthase-like, two probable beta-glucosidases, Beta-mannosidase b, and Ferric reductase NADH/NADPH oxidase and related proteins. This pattern is consistent with observations in *S. cerevisiae,* where cobalt stress induced genes involved in iron uptake and homeostasis, and of proteins involved in cell wall maintenance [65]. Moreover, transcriptomic profiling under cobalt stress in the yeast *D. hansenii* reported the upregulation of genes encoding proteins involved in cell wall repair [53], in line with our observation of carbohydrate-active enzymes, such as beta-glucosidase and beta-mannosidase, which may be associated with cell wall-related transcriptional responses. In addition, 85 genes were regulated under both hypoxic and CoCl_2_ treatments. Of these, 50 were downregulated, 11 were upregulated, and 24 showed opposite regulation. The log_2_FC of shared genes was generally comparable across treatments, although some showed stronger regulation under hypoxia. These included genes encoding High-affinity glucose transporter GHT2, Zinc finger HIT-type, Fumarate reductase, Pheromone-regulated membrane protein 10, Alternative oxidase mitochondrial, and Pkinase-domain-containing protein, all of which were more strongly downregulated under hypoxic conditions. In contrast, the Major facilitator-type transporter ecdD showed stronger upregulation under hypoxia. Among the genes with the most pronounced opposite regulation were a Probable beta-glucosidase, Lactose permease, and Xyl5_phach beta-xylosidase, which were downregulated under hypoxia but upregulated under CoCl_2_. Conversely, genes encoding DnaJ homolog 1 mitochondrial, DnaJ homolog subfamily B member 4, and Heat Shock Protein 90 homolog were upregulated under hypoxia but repressed by CoCl_2_ treatment. Together, these contrasting transcriptional patterns suggest that *P. rhodozyma* engages distinct gene expression programs in response to hypoxic versus CoCl_2_ exposure. Under oxygen limitation, repression of specific metabolic pathways and induction of molecular chaperones are consistent with a transcriptional profile associated with protein homeostasis, particularly in the mitochondria. In contrast, CoCl_2_ exposure is associated with transcriptional changes linked to nutrient utilization and detoxification-related processes, probably as part of a broader stress-responsive program.

### 3.3. Differential Transcriptomic Responses of the Δsre1 Mutant and the Wild-Type Under Hypoxia, CoCl_2_, and Clotrimazole Treatments

**Figure 3:**
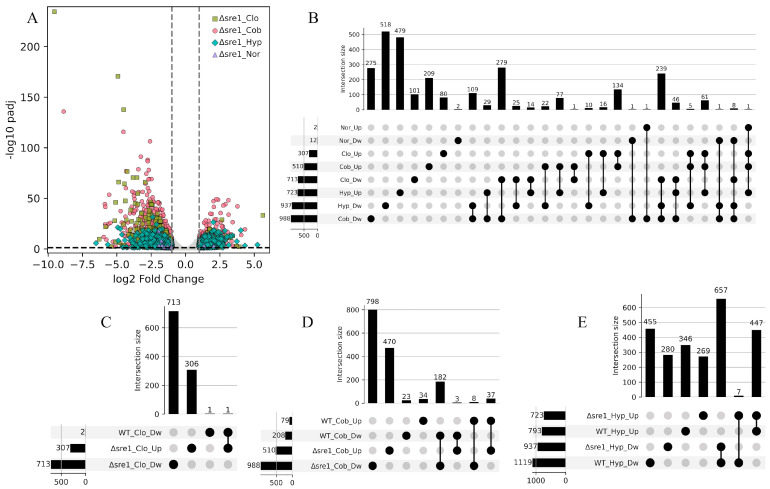
Transcriptional response of the *P. rhodozyma* Δ*sre1* mutant and wild-type (WT) under different treatments. Transcriptomes from normoxic (Nor), hypoxic (Hyp), cobalt chloride (Cob), and clotrimazole (Clo) conditions were compared with the untreated WT control. (**A**) Volcano plot of differentially expressed genes (DEGs) in the Δ*sre1* mutant. Significance thresholds are indicated by dashed lines (|log_2_FC| ≥ 1.0, *p*adj ≤ 0.05). (**B**) UpSet plot showing exclusive and shared downregulated (Dw) and upregulated (Up) DEGs among treatments in the Δ*sre1* mutant. (**C**–**E**) UpSet plots showing exclusive and shared DwDEGs and UpDEGs between the Δ*sre1* mutant and WT under each treatment.

To investigate the potential role of Sre1 in response to the three treatments, the transcriptomes of the Δ*sre1* mutant were determined under normoxic conditions and after exposure to hypoxia, CoCl_2_, or clotrimazole, and compared with the transcriptome of the untreated WT control. The transcriptional changes between the Δ*sre1* mutant and WT under normoxic conditions were minimal, with 12 DwDEGs and two UpDEGs (Figure 3). This observation suggests that the basal activity of Sre1 is associated with the maintenance of gene expression levels of a set of genes under untreated conditions, representing a core group of “basal Sre1-regulated genes”, consistent with the fact that Sre1 is activated at basal levels under this condition [35]. Among these genes, nine of the DwDEGs were previously identified as direct Sre1 targets by ChIP-Exo analysis, including the *SRE1* gene itself (g4728) [35] (Table 1). As expected, g4728 appears downregulated in the Δ*sre1* mutant under normoxic conditions, serving as an internal control. The other eight genes are related to ergosterol biosynthesis as they were assigned to the Sterol biosynthesis or Terpenoid backbone biosynthesis KEGG modules.

Unlike what was observed under untreated conditions, major changes were observed under the three treatments, with the strongest response under hypoxia (937 DwDEGs and 723 UpDEGs), followed by CoCl_2_ (988 DwDEGs and 510 UpDEGs) and clotrimazole (713 DwDEGs and 307 UpDEGs) (Figure 3A,B). In all cases, the number of DwDEGs exceeded the number of UpDEGs, a pattern consistent with that observed in the WT. In contrast to the WT treated under the same conditions, the Δ*sre1* mutant exhibited a higher number of DEGs in response to clotrimazole and CoCl_2_, but a reduced response to the hypoxic treatment. Across all conditions, the number of DEGs that were shared among treatments was greater in the Δ*sre1* than in the WT. The proportion of shared DEGs ranged from 45% to 86% for downregulated genes and from 34% to 74% for upregulated genes under hypoxic and clotrimazole treatments, respectively. The largest groups of shared DEGs corresponded to those downregulated under clotrimazole and CoCl_2_ (279 DEGs), downregulated across all three treatments (239 DEGs), and upregulated under clotrimazole and CoCl_2_ (134 DEGs). These results point to a stronger similarity in the transcriptomic response to clotrimazole and CoCl_2_ in the Δ*sre1* mutant.

When the direct Sre1 targets belonging to the core group of “basal Sre1-regulated genes” were examined, none of them showed significant changes in the WT under exposure to clotrimazole or CoCl_2_, and only one gene (g3516, Hydroxymethylglutaryl-CoA synthase) was downregulated in the WT under hypoxia. It is also important to note that the *SRE1* gene itself (g4728) did not show differential expression in the WT under any of the three treatments applied (Table 1). These observations indicate that, under the applied conditions, the treatments did not elicit transcriptional patterns consistent with robust activation of the SREBP pathway.

Therefore, most of the transcriptional changes observed in the Δ*sre1* mutant are likely attributable to the biological effects of the basal expression differences affecting the core group of “basal Sre1-regulated genes”, which appear to confer partial protection against the applied stress conditions in the WT, a protection that is lost in the Δ*sre1* mutant. Consequently, the transcriptional differences observed in the mutant, but not in the WT, can be attributed to the loss of a Sre1-mediated basal protection. Consistent with this, comparison of the Δ*sre1* and WT transcriptomes revealed DEGs exclusive to the mutant, including 713 downregulated and 306 upregulated under clotrimazole treatment, 798 downregulated and 470 upregulated under CoCl_2_ exposure, and 280 downregulated and 269 upregulated under hypoxic conditions (Figure 3C–E). Interestingly, under clotrimazole treatment, nearly all DEGs were detected exclusively in the Δ*sre1* mutant, suggesting that Sre1 in the WT is sufficient to confer resistance to clotrimazole, a function that is absent in the mutant, as previously reported [34]. A higher number of DEGs were shared between the mutant and the WT under CoCl_2_ and hypoxia, particularly among downregulated genes. Moreover, under hypoxia, the number of shared DEGs between the Δ*sre1* mutant and WT exceeded those exclusive to the mutant. These findings suggest that Sre1 is associated with a basal transcriptional buffering effect in the WT, which is more pronounced under CoCl_2_ stress than under low-oxygen conditions.

**Figure 4:**
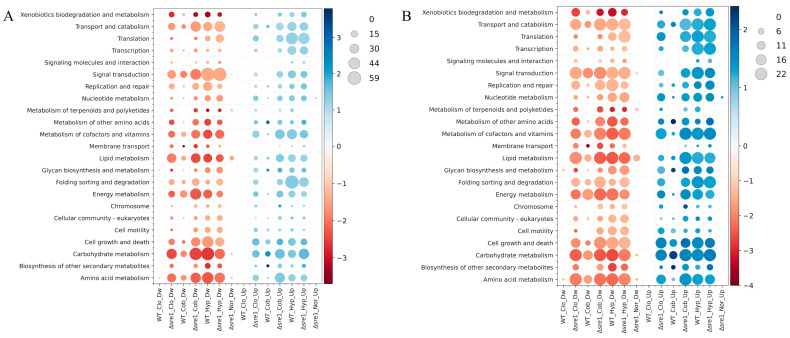
Differentially expressed genes across KEGG metabolic pathways and functional modules. (**A**) KEGG pathways and (**B**) KEGG modules. The heatmaps show the number of DEGs (circle size) and the average log_2_FC (circle color) for each category in the Δ*sre1* mutant and wild-type (WT) under normoxia (Nor), and clotrimazole (Clo), cobalt chloride (Cob), and hypoxia (Hyp) treatments.

Analysis of DEGs classified into KEGG metabolic pathways and modules (Figure 4A,B) revealed similar patterns across treatments in the Δ*sre1* mutant, with the clotrimazole and CoCl_2_ being once again the most similar. The top five pathways and modules with the highest number of DwDEGs across treatments included Carbohydrate metabolism, Amino acid metabolism, Signal transduction, and Transport and catabolism pathways, together with Other carbohydrate metabolism, Cofactor and vitamin metabolism, and Central carbohydrate metabolism modules. For UpDEGs, the most common top five categories were Carbohydrate metabolism and Metabolism of cofactors and vitamins pathways, and Cofactor and vitamin metabolism and Other carbohydrate metabolism modules. Major differences were also observed, particularly among DwDEGs assigned to Pyrimidine metabolism, and UpDEGs associated with the Biosynthesis of phytochemical compounds, Glycosaminoglycan metabolism, and Other amino acid metabolism, which were detected exclusively under hypoxic treatment. Notably, a substantial number of DwDEGs were assigned to the ATP synthesis module in response to CoCl_2_ treatment, with higher representation in the mutant. Under normoxic conditions, DwDEGs in the Δ*sre1* mutant were mainly classified within the Lipid metabolism and Metabolism of terpenoids and polyketides pathways, as well as the Sterol biosynthesis and Terpenoid backbone biosynthesis modules, consistent with the role of Sre1 in the upregulation of ergosterol biosynthetic genes [35]. Conversely, several pathways and modules were detected exclusively in the mutant but not in the WT (Figure 4A,B). Under clotrimazole treatment, these included 21 pathways and 17 modules among DwDEGs, mainly associated with Carbohydrate metabolism, Lipid metabolism, Signal transduction, Transport and catabolism, and Cofactor and vitamin metabolism. For UpDEGs, 21 pathways and 18 modules were identified, primarily related to Carbohydrate metabolism, Metabolism of cofactors and vitamins, Cell growth and death, and Central and Other carbohydrate metabolism. Under CoCl_2_ treatment, six pathways and four modules with DwDEGs were exclusively found in the mutant, including Cell motility, Biosynthesis of other secondary metabolites, Metabolism of terpenoids and polyketides, Fatty acid metabolism, and Sterol biosynthesis. The exclusive UpDEGs in the mutant were associated with eight pathways and 16 modules, mainly related to Translation, Signal transduction, and Folding sorting and degradation pathways, and Cofactor and vitamin metabolism and Central carbohydrate metabolism modules. Under hypoxia, mutant-exclusive DEGs were limited to three modules for UpDEGs, with a higher number of DEGs in Fatty acid metabolism. In general, when comparing pathways and modules shared between the Δ*sre1* mutant and the WT, a higher number of DwDEGs and UpDEGs was observed in the mutant under clotrimazole and CoCl_2_ treatments, while more similar numbers were detected under hypoxia. These observations are consistent with the limited overlap observed in transcriptomic studies of Δ*sre1* mutants and wild-type strains in *C. neoformans* subjected to different stressors. For example, under CoCl_2_ treatment, the WT displayed elevated expression of plasma membrane transporters, components of the electron transport chain, and enzymes associated with fatty acid, carbohydrate, and amino acid metabolism relative to the mutant. Such differential expression patterns were not observed between Δ*sre1* and wild-type strains under hypoxic conditions [17,26].

Overall, comparisons of DEGs across functional classifications revealed fewer transcriptional changes in the WT than in the Δ*sre1* mutant under CoCl_2_ and, particularly, under clotrimazole treatment. These observations are consistent with previous reports showing that the *P. rhodozyma* Δ*sre1* mutant is unable to grow in the presence of clotrimazole (0.15 µg/mL) or CoCl_2_ (400 µM), unlike the WT strain [34]. Therefore, a higher number of DEGs in the mutant compared to the WT under these treatments was expected. In contrast, under hypoxic conditions, the WT displayed a similar or even greater number of DEGs than the mutant across the KEGG metabolic pathways and modules. Taken together, these results indicate that basal Sre1 activity in the WT is associated with a reduced magnitude of transcriptional changes under clotrimazole and CoCl_2_ exposure, whereas under hypoxia, Sre1-dependent regulation alone is insufficient to account for the observed transcriptional responses. This suggests that hypoxia-responsive gene expression in *P. rhodozyma* likely involves additional regulatory components or pathways beyond basal Sre1 activity. This differs from what has been reported in other fungi. For example, in *A. fumigatus*, SrbA (the SREBP homolog) is essential for ergosterol and heme biosynthesis under hypoxia [31], and sterol biosynthesis has been described as an indirect oxygen-sensing process due to its strong oxygen dependence [51]. In *C. neoformans*, Sre1 regulates genes involved in sterol biosynthesis and hypoxia adaptation, and is required for growth under low-oxygen conditions [17,26]. Similarly, in *C. albicans,* the Zn(2)-Cys(6) transcription factor Upc2 is required for the induction of sterol biosynthetic genes and plays a key role in adaptation to hypoxia [12,15]. In line with these comparisons, our results suggest that under the conditions analyzed, hypoxia-induced transcriptional responses in *P. rhodozyma* are not fully explained by Sre1-dependent regulation alone.

### 3.4. Influence of Basal Sre1 Activity on Transcriptional Changes Under the Different Stress Conditions

**Figure 5:**
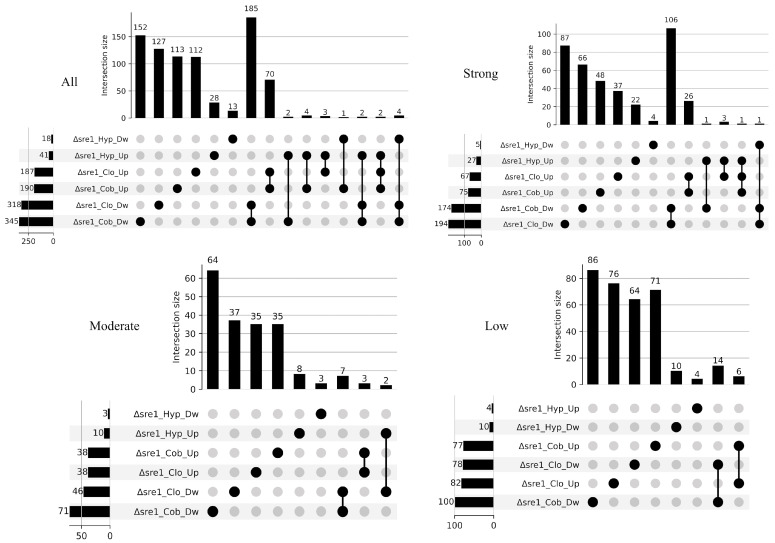
Comparison of genes exhibiting significantly greater transcriptional changes in the Δ*sre1* mutant than in the WT across clotrimazole (Clo), cobalt chloride (Cob), and hypoxia (Hyp) treatments. Analyses were conducted using all selected genes and were further classified into categories of strong (|log_2_FC| ≥ 1.5), moderate (1.3 > |log_2_FC| ≥ 1.0), and low (|log_2_FC| < 1.0) transcriptional changes.

**Figure 6:**
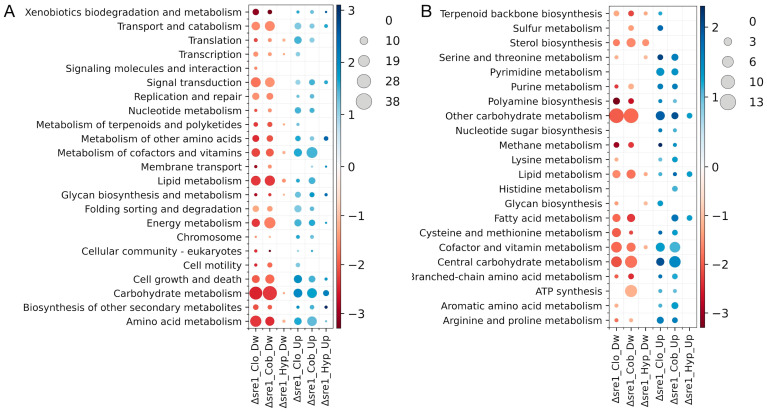
Classification of genes significantly expressed in the Δ*sre1* mutant versus WT under different treatments. (**A**) KEGG pathways, (**B**) KEGG modules. The heatmaps show the number of DEGs (circle size) and the average log_2_FC (circle color) for each category in the Δ*sre1* mutant under (Clo), cobalt chloride (Cob), and hypoxia (Hyp) treatments.

To investigate the influence of basal Sre1 activity and of the core group “basal Sre1-regulated genes” on transcriptional responses to the different treatments, we focused on genes whose expression significantly changed in the mutant compared to the WT. This expression pattern suggests that basal Sre1 activity in the WT may contribute to buffering transcriptional changes induced by the treatments, an effect that is lost in the mutant. These genes include those that were unchanged in the WT but differentially expressed in the Δ*sre1* mutant, as well as genes that changed in the same direction in both strains but with a greater magnitude of change in the mutant. In addition, to have a clear view of the transcriptional changes, genes without BLASTp hits or with hypothetical proteins were excluded. Applying these criteria, a higher number of genes was detected in the CoCl_2_ and clotrimazole treatments (535 and 505, respectively), whereas a lower number was observed under hypoxia (59 genes; Figure 5). This distribution indicates that the absence of basal Sre1 activity is associated with more pronounced transcriptional changes under clotrimazole and CoCl_2_ treatments, and to a lesser extent under hypoxic conditions. Among all selected genes, the highest number of shared genes was observed between the cobalt and clotrimazole treatments, and the lowest between the hypoxic treatment and the others. Notably, the majority of genes shared between the cobalt and clotrimazole treatments correspond to a group of strongly regulated genes, particularly those that are downregulated. Consistently, when the selected genes were classified into metabolic pathways and functional modules, major similarities were observed between CoCl_2_ and clotrimazole treatments, with a higher number of pathways (22 and 23, respectively) and modules (15 and 17, respectively), in which both down- and upregulated genes were classified (Figure 6). As can be observed, the pathways in which a high number of genes were classified correspond to core cellular processes and global metabolic functions, such as Carbohydrate metabolism, Amino acid metabolism, Signal transduction, Lipid metabolism, and Metabolism of cofactors and vitamins. These results indicate that, in the absence of basal Sre1 activity, clotrimazole and CoCl_2_ treatments are associated with broader transcriptional reprogramming in the Δ*sre1* mutant, whereas such changes are less pronounced in the WT. This effect was particularly evident under clotrimazole treatment. Furthermore, genes were also classified into more specific pathways, such as Biosynthesis of other secondary metabolites, Glycan biosynthesis and metabolism, Metabolism of terpenoids and polyketides, and Xenobiotic biodegradation and metabolism, which may be involved in more specific responses to these compounds. Among the identified genes (Table S3), some were reported to be upregulated in the opportunistic pathogen *A. fumigatus* exposed to azoles, including glutathione S-transferase, Glutathione S-transferase family protein, several putative cytochrome P450 monooxygenases, and an aldehyde reductase II [66]. An upregulation of Glutathione S-transferase, dependent on Sre1, was reported in the pathogenic fungus *C. neoformans* treated with 0.6 mM CoCl_2_ [17]. An increase in azole susceptibility of *A. fumigatus* by deletion of alleles of two different ABC transporter-encoding genes in three different strains suggested a contribution of ATP-Binding Cassette Transporter Proteins to drug resistance in this fungus [67]. Other detected genes encoded sterol 14α-demethylase (Cyp51), a cytochrome P450 (CYP) enzyme required for sterol biosynthesis in various phyla, in which transcriptional regulation mechanisms have been studied mainly in mammalian cells, being regulated primarily by the cholesterol level through SREBP, a regulation that has also been stated to be highly conserved in fungi [68].

Therefore, the transcriptomic changes observed in the absence of Sre1 basal activity involve both global cellular aspects and others directly related to the SREBP pathway, including direct gene targets of Sre1.

### 3.5. Direct Sre1 Targets Under the Different Stress Conditions

As previously stated, the genes differentially expressed under the treatments are likely attributable to the biological effects of the basal Sre1 activity acting on the core group of “basal Sre1-regulated genes”. In previous work, ChIP-exo experiments identified putative direct Sre1 targets in *P. rhodozyma* [35], which were analyzed among DEGs in the Δ*sre1* mutant relative to WT under all three treatments. All of these potential direct Sre1 targets were differentially expressed only in the Δ*sre1* mutant under all the tested conditions, except for a single gene (g3516), which was differentially expressed in the WT, but to a lesser extent than in the mutant under hypoxia. These observations support an association between basal Sre1 activity in the WT and the maintenance of expression of these genes under the applied stresses, an association that is lost when Sre1 is absent.

Once again, the transcriptional pattern under hypoxia differs from that under the other treatments, with the Δ*sre1* mutant showing fewer differentially expressed genes than WT. The hypoxia treatment had only three potential Sre1 target genes in common with the other treatments and two exclusive genes (g943 and g5758) that were upregulated. The upregulation of these two genes (encoding NAD(P)-dependent alcohol dehydrogenase and ATP-dependent permease, respectively) may reflect transcriptional adjustments related to redox balance and transport processes under low-oxygen conditions. Together, these observations suggest that Sre1-dependent transcriptional regulation is less prominent under the applied hypoxic conditions, or alternatively, that the hypoxic treatment used here was insufficient to elicit a distinct Sre1-associated transcriptional signature.

Seven of the potential direct Sre1 target genes (g190, g904, g1347, g1377, g3516, g3611, and g5928) encode key enzymes of the ergosterol biosynthetic pathway. Three of them (g904, g1347, and g3516) were downregulated in the Δ*sre1* mutant under all three treatments. Gene g1377 was downregulated under clotrimazole and CoCl_2_, two genes (g190 and g3616) were downregulated exclusively under CoCl_2,_ and gene g5828 was downregulated exclusively under clotrimazole (Table 2). Within this group of genes, two of them encode enzymes of the mevalonate pathway (g3516, Hydroxymethylglutaryl-CoA Synthase, and g1317, Hydroxymethylglutaryl-CoA reductase), which provide the precursors required for ergosterol biosynthesis in *P. rhodozyma* [69]. The gene g5928 encodes a Cytochrome P450 reductase, which donates electrons to cytochrome P450 enzymes, two of which are involved in ergosterol biosynthesis. Downregulation or mutation of this type of reductase has been associated with increased azole sensitivity [34,70,71], which is consistent with its downregulation under clotrimazole treatment in the Δ*sre1* mutant. In this context, gene g190 encodes a Lanosterol 14-alpha demethylase, which is a cytochrome P450 (Cyp51). The lanosterol 14-alpha demethylase is the primary target of azole antifungals, and its upregulation is a known mechanism of resistance towards these types of compounds [72]. Then, its downregulation in the Δ*sre1* mutant under CoCl_2_ is consistent with altered transcriptional regulation of sterol biosynthetic genes in the absence of Sre1. Gene g3611 encodes Lanosterol synthase (Erg7), which catalyzes the formation of lanosterol, the first committed intermediate of the sterol pathway. The downregulation of *ERG7* under CoCl_2_ in the Δ*sre1* mutant further supports the idea that Sre1 contributes to sustain early steps of sterol biosynthesis during stress. A reduction in lanosterol production would be expected to impact the downstream reactions needed to restore sterol levels. The gene g1347 encodes Delta 14-sterol reductase (Erg24), which catalyzes a reduction step post-lanosterol production of the ergosterol pathway. In *A. fumigatus*, two *ERG24* paralogs have been identified, in which a conditional double mutant showed defects in ergosterol biosynthesis and growth. The severe growth defect in this mutant could be rescued by supplementation of metal ions or by overexpressing ion transporters, indicating that Erg24 activity is associated with ion homeostasis [73]. Related to this, the gene g4256, which encodes a potential Cation diffusion facilitator family transporter, was downregulated exclusively in the mutant under CoCl_2_ treatment. Therefore, the downregulation of g1347 in the Δ*sre1* mutant under clotrimazole and CoCl_2_ and of g4256 under CoCl_2_ is consistent with a reduced capacity to maintain sterol and membrane homeostasis in the absence of Sre1. Finally, the gene g904 encodes a Sterol 24-C-methyltransferase, which generally catalyzes the conversion of zymosterol to fecosterol, which occurs several steps downstream of lanosterol production [74]. However, in basidiomycetes such as *C. neoformans*, this enzyme also participates in an alternative pathway that converts lanosterol into eburicol, also leading to ergosterol, and its inhibition disrupts ergosterol homeostasis [75]. In this alternative pathway*,* Sterol 24-C-methyltransferase functions upstream of the azole target Lanosterol 14-alpha demethylase (Cyp51), highlighting its relevance in azole-related antifungal responses [76]. Taken together, these patterns are consistent with the idea that basal Sre1 activity supports multiple points of the ergosterol pathway, from the initial production of lanosterol to later modification steps, thereby contributing to membrane and sterol homeostasis during stress.

In addition, the downregulation of carbohydrate-modifying enzymes encoding genes, such as Glycosyltransferase family 25 (g806), Glycoside hydrolase superfamily (g5034), L-rhamnonate dehydratase (g1192), and Alpha-amylase 1 (g5613), in the Δ*sre1* mutant under clotrimazole and CoCl_2_ exposure suggests a broader association between basal Sre1 activity and transcriptional programs related to carbon metabolism during stress. This interpretation is consistent with the KEGG-based functional classification of DEGs, where Carbohydrate metabolism, Other carbohydrate metabolism, and Central carbohydrate metabolism were among the most represented categories in the mutant under both treatments (Figure 6). Omics studies in *Candida* and other yeasts have shown that antifungal treatment frequently reshapes metabolic pathways being carbohydrate metabolism consistently affected [77], and the Sre1-dependent changes observed in this work are in line with such a metabolically centered adaptation to clotrimazole and CoCl_2_ stress. The reduced expression of these genes in the absence of Sre1 may indicate an impaired ability to redirect carbon fluxes under stress, thereby limiting the metabolic flexibility required for an effective response to clotrimazole and CoCl_2_. Interestingly, g750 was upregulated in the Δ*sre1* mutant under CoCl_2_. This gene encodes ATP-citrate lyase, which plays a key role linking carbohydrate metabolism to lipid synthesis. This enzyme converts citrate into acetyl-CoA and oxaloacetate, and acetyl-CoA is required for fatty acid and sterol biosynthesis [78]. Then, even though this gene may represent a direct Sre1 target, its upregulation in the Δ*sre1* mutant under CoCl_2_ likely reflects Sre1-independent compensatory transcriptional regulation.

It is also noteworthy that gene g944, which encodes an RTA1 domain-containing protein, was upregulated under both clotrimazole and CoCl_2_, and that gene g2548, which encodes an MFS general substrate transporter, was upregulated under CoCl_2_. RTA1-domain-containing proteins have been implicated in antimicrobial resistance and secretion-related functions in *C. neoformans* [79], and the upregulation of genes encoding members of the Major Facilitator Superfamily (MFS) has been associated with azole resistance in *C. albicans* [80]. Therefore, although g944 and g2548 may represent direct Sre1 targets, their upregulation in the Δ*sre1* mutant in clotrimazole and CoCl_2_ treatments likely reflects compensatory transcriptional mechanisms operating independently of Sre1.

## 4. Conclusions

Comparative transcriptomic analysis revealed that, under the tested conditions, loss of Sre1 in *P. rhodozyma* had only a minor impact on the early hypoxic transcriptional response, suggesting that Sre1 is not a dominant regulator of hypoxia-associated gene expression during short-term oxygen limitation in this yeast. In contrast, clear Sre1-associated transcriptional changes were detected under clotrimazole treatment and, to a lesser extent, following CoCl_2_ exposure, predominantly affecting genes related to membrane-associated processes and central metabolism. These patterns are consistent with the mode of action of the applied treatments, as clotrimazole directly targets sterol biosynthesis, whereas hypoxia and CoCl_2_ are expected to influence lipid metabolism more indirectly and in a time-dependent manner. Despite the limitations imposed by the sensitivity of the Δ*sre1* mutant to these treatments, the short-term treatments used in this study allowed for the identification of putative Sre1-associated transcriptional responses. Together, these results suggest that in *P. rhodozyma*, Sre1 plays a more prominent role in transcriptional responses to perturbations of sterol biosynthesis than in the early hypoxic response. These findings provide a framework for future studies exploring a broader range of treatment conditions and growth states, combined with targeted gene expression analyses and complementary functional approaches, to further clarify the role of Sre1 in stress-associated transcriptional regulation in this yeast.

## Figures and Tables

**Table 1:** Direct Sre1 targets differentially expressed in the Δ*sre1* mutant versus the WT under normoxic conditions, and their log_2_ fold change values in the WT under each treatment.

		Δ*sre1*_Nor		WT_Clo		WT_Cob		WT_Hyp	
Gene	Protein (KEGG Module Level 2)	log_2_	*p*adj	log_2_	*p*adj	log_2_	*p*adj	log_2_	*p*adj
g1347	Delta 14-sterol reductase, Erg24 (Sterol biosynthesis)	−1.8	7.4 × 10^−9^	0.2	1.0 × 10^0^	0.0	9.8 × 10^−1^	0.2	7.1 × 10^−1^
g1377	Hydroxymethylglutaryl-CoA reductase (Terpenoid backbone biosynthesis)	−1.0	5.3 × 10^−3^	0.0	1.0 × 10^0^	−0.2	8.2 × 10^−1^	−1.2	3.0 × 10^−6^
g190	Lanosterol 14-alpha demethylase (Sterol biosynthesis)	−1.1	3.2 × 10^−2^	0.2	1.0 × 10^0^	−0.4	6.9 × 10^−1^	0.8	5.8 × 10^−2^
g3516	Hydroxymethylglutaryl-CoA synthase (Terpenoid backbone biosynthesis)	−1.5	1.6 × 10^−2^	0.4	1.0 × 10^0^	0.5	6.5 × 10^−1^	−1.3	1.2 × 10^−2^
g3611	Lanosterol synthase (Sterol biosynthesis)	−1.3	7.5 × 10^−4^	0.1	1.0 × 10^0^	0.0	9.7 × 10^−1^	−0.5	4.5 × 10^−1^
g5794	Delta(7)-sterol 5(6)-desaturase, Erg3 (Sterol biosynthesis)	−1.0	3.9 × 10^−2^	−0.1	1.0 × 10^0^	−0.2	7.2 × 10^−1^	0.5	2.2 × 10^−1^
g602	Methylsterol monooxygenase, Erg25 (Sterol biosynthesis)	−1.6	3.9 × 10^−2^	0.0	1.0 × 10^0^	−0.3	7.1 × 10^−1^	0.0	9.8 × 10^−1^
g904	Sterol 24-C-methyltransferase (Sterol biosynthesis)	−1.9	1.7 × 10^−4^	0.5	1.0 × 10^0^	0.6	5.6 × 10^−1^	−0.8	2.6 × 10^−1^
g4728	Sterol regulatory element binding protein	−2.0	1.5 × 10^−18^	−0.1	1.0 × 10^0^	−0.2	7.8 × 10^−1^	−0.9	1.8 × 10^−4^

**Table 2:** Direct Sre1 targets that showed differential expression in the Δ*sre1* mutant versus the WT under the same conditions.

		Δ*sre1* vs. WT (Hyp)		Δ*sre1* vs. WT (Cob)		Δ*sre1* vs. WT (Clo)	
Gene	BLASTp Hit	log_2_	*p*adj	log_2_	*p*adj	log_2_	*p*adj
g190	Lanosterol 14-alpha demethylase, Cyp51			−1.6	1.5 × 10^−5^		
g750	ATP-citrate lyase			1.7	2.2 × 10^−6^		
g806	Glycosyltransferase family 25			−3.8	1.0 × 10^−5^	−4.3	3.6 × 10^−65^
g904	Sterol 24-C-methyltransferase	−1.6	2.7 × 10^−2^	−2.5	1.5 × 10^−8^	−1.9	2.3 × 10^−9^
g943	NAD(P)-dependent alcohol dehydrogenase	2.2	6.3 × 10^−3^				
g944	RTA1 domain-containing protein			2.1	4.4 × 10^−9^	3.0	3.0 × 10^−12^
g1192	L-rhamnonate dehydratase			−2.4	1.1 × 10^−38^	−1.7	2.3 × 10^−8^
g1193	Hypothetical protein_g1193			−2.3	5.3 × 10^−6^	−1.5	3.7 × 10^−3^
g1347	Delta(14)-sterol reductase (Erg24)	−1.8	3.7 × 10^−3^	−1.4	3.8 × 10^−9^	−1.3	7.6 × 10^−6^
g1377	Hydroxymethylglutaryl-CoA reductase			−1.5	8.6 × 10^−10^	−1.1	3.9 × 10^−6^
g2548	MFS general substrate transporter			1.9	5.4 × 10^−19^		
g3516 *	Hydroxymethylglutaryl-CoA Synthase	−1.3	2.4 × 10^−2^	−2.9	4.4 × 10^−13^	−1.5	7.8 × 10^−5^
g3611	Lanosterol synthase (Erg7)			−1.3	3.4 × 10^−6^		
g4112	Hypothetical protein_g4112					−1.4	6.8 × 10^−10^
g4256	Cation diffusion facilitator family transporter/Metal tolerance protein			−1.6	8.3 × 10^−4^		
g5034	Glycoside hydrolase superfamily			−1.2	5.4 × 10^−7^	−1.5	1.3 × 10^−6^
g5613	Alpha-amylase			−3.5	1.3 × 10^−17^	−2.2	4.3 × 10^−6^
g5758	ATP-dependent permease	1.9	2.2 × 10^−2^				
g5928	Cytochrome P450 reductase					−1.3	3.6 × 10^−5^

All genes included in this Table were exclusively differentially expressed in the Δ*sre1* mutant under the conditions tested, except for g3516 (indicated with an *), which was differentially expressed in the WT, but to a lesser extent than in the mutant under hypoxia. Clotrimazole (Clo), cobalt chloride (Cob), and hypoxia (Hyp) treatments.

## Data Availability

The datasets generated and analyzed during this study are available at the National Center for Biotechnology Information BioProject: PRJNA966154 and as supplementary materials.

## Supplementary Materials

The following supporting information can be downloaded at https://www.mdpi.com/article/10.3390/jof12030200/s1, Table S1. FASTQ file statistics and quality control summary. Table S2. Log_2_ fold change strain1_condition versus strain2_condition and BLASTp hits. Table S3. Genes exhibiting significantly greater transcriptional changes in the Δ*sre1* mutant than in the WT across treatments.

## Abbreviations

The following abbreviations are used in this manuscript:

Clo	Clotrimazole
Cob	Cobalt chloride, CoCl_2_
DEGs	Differentially Expressed Genes
DwDEGs	Downregulated Differentially Expressed Genes
ER	Endoplasmic Reticulum
GO	Gene Ontology
Hyp	Hypoxia
KEGG	Kyoto Encyclopedia of Genes and Genomes
Nor	Normoxia
SRE	Sterol Regulatory Element
SREBP	Sterol Regulatory Element-Binding Protein
UpDEGs	Upregulated Differentially Expressed Genes
WT	Wild-type
